# Thomson–Einstein’s Tea Leaf Paradox Revisited: Aggregation in Rings

**DOI:** 10.3390/mi14112024

**Published:** 2023-10-30

**Authors:** Kirill Kolesnik, Daniel Quang Le Pham, Jessica Fong, David John Collins

**Affiliations:** 1 Department of Biomedical Engineering, University of Melbourne, Melbourne, VIC 3010, Australia; k.kolesnik@unimelb.edu.au (K.K.); jessica.fong@unimelb.edu.au (J.F.); 2 The Graeme Clark Institute, The University of Melbourne, Parkville, VIC 3010, Australia

**Keywords:** tea leaf paradox, micromanipulation, stokes drag, hydrodynamic interactions, computational fluid dynamics

## Abstract

A distinct particle focusing spot occurs in the center of a rotating fluid, presenting an apparent paradox given the presence of particle inertia. It is recognized, however, that the presence of a secondary flow with a radial component drives this particle aggregation. In this study, we expand on the examination of this “Thomson–Einstein’s tea leaf paradox” phenomenon, where we use a combined experimental and computational approach to investigate particle aggregation dynamics. We show that not only the rotational velocity, but also the vessel shape, have a significant influence on a particle’s equilibrium position. We accordingly demonstrate the formation of a single focusing spot in a vessel center, as has been conclusively demonstrated elsewhere, but also the repeatable formation of stable ring-shaped particle arrangements.

## 1. Introduction

**Figure 1:**
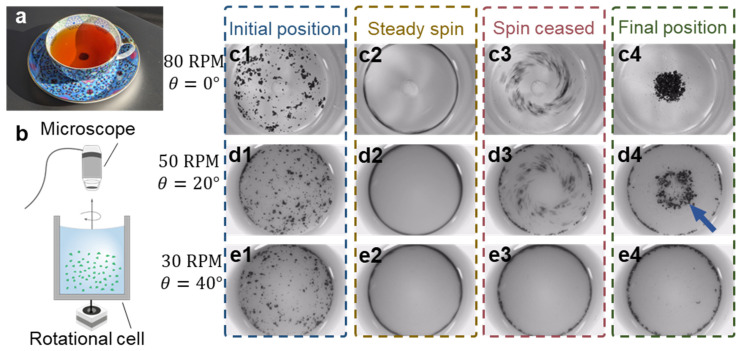
Tea leaf paradox for particle manipulation. (**a**) Tea leaf focusing in a cup after being stirred. (**b**) Schematic figure of the experimental setup. (**c**–**e**) Experimental results depicting distinct tea leaf patterns (top-down view), including (**c**) dot, (**d**) ring, and (**e**) edge particle aggregation governed by the vessel and rotation parameters. The subfigures illustrate the temporal evolution of the aggregation process: (**c1**–**e1**) initial distribution of tea leaves, (**c2**–**e2**) particles accumulating at the vessel’s edge during constant rotation, (**c3**–**e3**) particle movement induced by secondary flow immediately after the rotation ceases, (**c4**–**e4**) final distribution of tea leaves.

The tea leaf paradox is a well-known hydrodynamic phenomenon which notes the counterintuitive aggregation of particles in the middle of a rotated cup (Figure 1a). Indeed, if these particles are suspended in the rotating fluid, they experience greater centrifugal force than the surrounding fluid, which should move them towards the vessel edge, serving as the basis for this apparent paradox. While the study of this phenomenon is often attributed to Einstein due to his work on the topic [1], it was first interrogated by James Thomson in 1857 [2], who noted the appearance of a secondary flow in the radial direction. This effect arises from viscous friction near the stationary surface which generates an imbalance between the velocity and radial pressure in the boundary layer, causing a secondary flow transverse to the primary circulation. Gravitationally sedimented tea leaves can then be translated due to the secondary flow along a vessel’s base and focused in its center, rather than at the vessel edges. This secondary-flow-induced particle concentration has since been usefully applied to microcapsule scaffold concentration [3], dynamic circulation of charged dust particles [4,5], and blood plasma separation [6].

The tea leaf paradox can be further understood and generalized in the context of three-dimensional fluid systems, where the primary flow is often complemented with an orthogonal secondary flow which commonly arises due to the drag in the boundary layers. Flow in a curved channel, for example, generates secondary vortices which were reported to be adapted for hydrodynamic focusing and sorting [7]. Another example of secondary flow is the Taylor–Couette flow, which features counter-rotating stable vortices in the gap between a rotating cylinder and a static shell and is widely used in (bio-)chemical reactors [8,9]. Spatial control over cells and particles is required in many biochemical studies including cell analysis, solid-phase chemistry, and molecular assembly. Hydrodynamic forces generally show great potential for tailored micromanipulation. The lift force in shear flow allows for the rapid exposure of cells to a reacting solution [10]. Hydrodynamic forces have also been adapted for precise trapping and two-dimensional manipulation of micron-scale particles in the microfluidic environment [11]. The integration of hydrodynamic forces with other forces can further enhance micromanipulation activities, opening new possibilities for advanced applications. For example, the combined effect of the hydrodynamic, centrifugal and acoustophoretic forces has been employed to concentrate particles in sessile droplets on piezoelectric substrates [12,13].

In the case of the tea leaf paradox, this has been typically appropriate for concentration activities, resulting in the formation of a single particle cluster at the center of the flow system. However, the influence of systematically modifying the bottom surface of a vessel remains unexplored. In this study, we demonstrate that this modification can result in further non-intuitive outcomes, including the formation of ring structures, indeed at the edges of the vessel, instead of solely aggregation in the vessel center. These equilibrium positions are achieved through control of the geometry and actuation conditions, which we parametrically investigate in both experiments and simulations. We further present a numerical approach to analyze driving forces and predict particle sedimentation in axisymmetric domains with a secondary axial flow. This generalized approach can be also used to model the sedimentation behavior of particles in vessels with a secondary axial flow for other particle manipulation activities, with implications for the control of particle aggregation and sedimentation in various fields including biotechnology, chemical engineering, and materials science.

## 2. Methods

### 2.1. Computational Fluid Dynamics

The study employs Navier–Stokes equations to model the motion of viscous fluid numerically. Specifically, for an incompressible single-phase Newtonian fluid, the momentum and continuity equations are expressed as follows:

(1) $$\rho_{f}\left(\frac{\partial u}{\partial t}+\left(u\cdot \nabla \right)u\right)=-\nabla p+\nabla \cdot \mu_{f}\left(\nabla u+{\left(\nabla u\right)}^{T}\right)\;\rho_{f}\nabla \cdot u=0$$

where u denotes the fluid velocity, p is the pressure, $\rho_{f}$ is the density, and $\mu_{f}$ is the dynamic viscosity [14] (see Table S1). The computational domains are shown in Figure S1 where the diameter is $D=2R_{0}=30$ (mm), and the height is H=40 (mm); a positive inclination angle θ indicates a concave shape, and a negative angle indicates a convex shape (Figure S2a,b). The axisymmetric flow can be characterized by a rotational (circumferential) Reynolds number:

(2) Reφ=ρfωR02μf.

Implying the initial rotational rate $\omega_{0}$, the Reynolds number ranges from 800 to 2100 at the rotational rates 30–80 (RPM).

In the initial state (t=−0.1 s), the fluid is assumed to rotate with a constant angular velocity $\omega_{0}$ around the axis of symmetry (Z)

(3) $$u_{\varphi }=R\omega_{0},$$

where R is the distance from the rotational axis; and $\omega_{0}$ denotes the initial angular velocity. The vessel boundaries at the initial frame (t=−0.1 s) have an identical rotational rate as the fluid. Over a period of 0.2 s (from −0.1 s to 0.1 s), the domain boundaries decelerated and came to a stop. A no-slip boundary condition is applied to the side and bottom boundaries of the fluid domain (Figure S2c)

(4) $$u_{\varphi }\left(t\right)=R\omega_{0}\left(1-H\left(t\right)\right),$$

where H(t) is a smoothed unit step function, enabling a smooth transition from the vessel rotation to a state of rest

(5) $$H\left(t\right)=0,\;if\;t\leq 0\;H\left(t\right)=1,\;if\;t\geq 0.2\;H\left(t\right)=0.5+0.75\left(\frac{t-0.1}{0.1}\right)-0.25{\left(\frac{t-0.1}{0.1}\right)}^{3}$$

At the top of the computational domain, a slip boundary condition is applied to define a free surface

(6) u·n=0,

where n denotes the unit vector normal to the plane. In general, when a liquid rotates with a constant angular velocity $\omega_{0}$, it creates a parabolic-shaped free surface. The maximum elevation of this surface for a vessel with diameter D can be calculated as

(7) $$\delta =\frac{\omega_{0}{}^{2}D^{2}}{8g},$$

where g is the acceleration of gravity. However, in our study, the highest angular velocity used, $\omega_{0}=80$ RPM, which results in a relatively small elevation δ≈0.8 (mm), which is negligible compared to the height of the domain. Therefore, for simplicity, we assume a flat top surface for the computational domain.

The Computational Fluid Dynamics (CFD) simulations were performed using the COMSOL Multiphysics 5.5 software, which utilizes the Finite Element Method (FEM) for solving the governing equations. The model was implemented using a time-dependent single-phase laminar flow (SPF) module. The simulations compute the system evolution from −0.1 s to 90 s, where the smoothed rotation termination is centered at the 0-time frame. A MATLAB routine is used to execute the parametric solver and process the resulting data.

### 2.2. Computational Mesh Study

The structured mesh employed in this study is depicted in Figure S4a. To address the issue of highly skewed cells, an O-grid technique was implemented to discretize circular cross-sections. To ensure accuracy and capture the viscous boundary layer, the mesh was refined near the side and bottom walls. The color table in Figure S2b represents the skewness of the mesh elements

(8) $$S=1-max\left(\left|\frac{2\xi }{\pi }-1\right|\right),$$

where S=1 represents a perfectly regular element and S=0 represents a degenerated element, ξ is the angle over an edge in the element, and the maximum is taken over all edges of the element.

To assess the mesh convergence, this study follows the approach of Devendran et al. [15] and Muller et al. [16]. Figure S2b plots the convergence function

(9) $$C\left(g\right)=\sqrt{\frac{\int^{\;}{\left(u-u_{ref}\right)}^{2}dx\;dy}{\int^{\;}{\left(u_{ref}\right)}^{2}dx\;dy}}$$

where u is the current solution; and $u_{ref}$ is a reference solution with a maximum mesh element size of $d_{mesh\;ref}=0.8$ (mm). The analysis was performed at a rotational velocity of $\omega_{0}=60$ (RPM) and a base inclination angle of θ=0. All components of the speed were analyzed independently. The threshold for mesh convergence was set C(g)<0.01, corresponding to a maximum mesh size of $d_{mesh}=1.4$ (mm).

### 2.3. Particle Modeling

The particle sedimentation process is modeled based on the results of a time-dependent CFD analysis. Since we study particles that are heavier than water, we examine the velocity profiles in the bottom 5 mm thick region of the domain (see Figure S2c). The velocity components in the xz-planes are averaged over 40 equidistant φ values. To predict particle aggregation, their motion was decoupled into out-of-plane (circumferential) and in-plane (xz plane) motion. 

To estimate the forces acting on the particle, we consider it as a spherical particle, quasi-static in the rotating xz-frame (Figure S2d). The local coordinate system rotates around the vessel axis with an angular velocity corresponding to the out-of-plane fluid velocity $u_{\varphi }$ at the particle location in the xz-plane. This setup simulates a particle with a circular trajectory around the axis. The forces along *z* and *x* axes are decoupled since particle displacement in these coordinates has different order of length scales. 

In the first step, we calculate particle separation distance from the bottom surface (along *z*-axis). We assume that the particles are located in a close proximity to the bottom of the vessel. Due to the forces counteracting the gravitational sediment, particles can move along the vessel bottom. The total force on a particle along *z*-axis is the combination of the lift force, drag force, centrifugal force, and gravitational force projections, with

(10) $$F_{z}=F_{lift,z}+F_{drag,z}+F_{cent,z}+F_{g,z}.$$

For a particle with an effective particle radius in a shear velocity field, this experiences a lift force according to the following approximated expression [17]:

(11) $$F_{lift,z}=6.46\rho_{f}\sqrt{\nu_{f}}r_{p}^{2}u_{x}\sqrt{\left|\frac{du_{x}}{dz}\right|}\mathrm{sign}\left(\frac{du_{x}}{dz}\right)$$

where $\nu_{f}=\mu_{f}/\rho_{f}$ is the kinematic viscosity of the water, $\mu_{f}$ is the viscosity of the fluid, u is the fluid velocity at the particle center, and the sign function extracts the sign of a quantity. The drag force acting on the particle due to z fluid component is given by

(12) $$F_{drag,z}=6\pi \mu_{f}r_{p}u_{z}.$$

Further, the projection of the centrifugal force projection can be derived as follows, with

(13) $$F_{cent,z}=\frac{4}{3}\pi r_{p}^{3}\left(\rho_{p}-\rho_{f}\right)\frac{u_{\varphi }^{2}}{R}sin\theta ,$$

where *R* is the distance from the rotational axis, $u_{\varphi }$ is the out-of-plane fluid velocity, and θ is the bottom inclination angle. The gravitational force projection on the z-axis is defined as

(14) $$F_{g,z}=\left(\rho_{f}-\rho_{p}\right)\frac{4}{3}\pi r_{p}^{3}gcos\theta ,$$

where $\rho_{f}$ is the fluid density, $\rho_{p}$ is the particle density, $r_{p}$ is the effective particle radius, and g is gravitational acceleration. The total force acting on the particle is evaluated at a given point above the surface. If the total force is pointing downwards everywhere ($F_{z}\leq 0$), the particle is considered to be in contact with the surface. Otherwise, by imposing the condition $F_{z}=0$, the equilibrium particle elevation can be calculated. As the lift force rapidly decays away from the surface, the particle equilibrium position is $\approx r_{p}$. 

In the second step, the x-force component is evaluated taking into account the equilibrium particle elevation along z-axis. When a particle is placed in a shearing flow near a plane wall, the classic Stokes drag is no longer valid since the nearby wall induces alterations in the fluid flow field around the particle. We therefore employ a modified Stokes formula [18], with

(15) $$F_{drag,x}=6\pi \mu_{f}r_{p}u_{x}F_{x}^{s*}\left(z\right),$$

where $F_{x}^{s*}\left(z\right)$ is a compensation factor depending on the distance from the surface (see Table S3) and reaches $F_{x}^{s*}\left(r_{p}\right)=1.7005$ for a sphere adjacent to the surface. The centrifugal and gravitational force components are given by

(16) $$F_{cent,x}=\frac{4}{3}\pi r_{p}^{3}\left(\rho_{p}-\rho_{f}\right)\frac{u_{\varphi }^{2}}{R}cos\theta ,$$

(17) $$F_{g,x}=\left(\rho_{p}-\rho_{f}\right)\frac{4}{3}\pi r_{p}^{3}gsin\theta .$$

Particles in contact with the boundary experience the solid friction force which is proportional to the normal force component

(18) $$F_{fric,x}=\mu \left|F_{z}\right|,$$

where μ is the solid friction constant. The total force along the local *x*-axis is determined as follows, with

(19) $$F_{x}=\{\begin{array}{cc} 0 & if\;F_{fric,x}\geq \left|{\tilde{F}}_{x}\right|\\ \left(\left|{\tilde{F}}_{x}\right|-F_{fric,x}\right)\;\mathrm{sign}\left({\tilde{F}}_{x}\right) & if\;F_{fric,x}<\left|{\tilde{F}}_{x}\right| \end{array}\;{\tilde{F}}_{x}=F_{drag,x}+F_{cent,x}+F_{g,x}$$

Figure S3a–c shows the dimensionless velocity components in the local coordinate system 5 s after the termination. The fluid flow has a dominant out-of-plane $u_{\varphi }$ component which governs the circumferential particle motion. In-plane components of the fluid flow, however, exhibit lower amplitude in general, though with highest velocities in the adjacent layer to the bottom surface. Figure S3d plots the evolution of the resulting force $F_{x}$, acting on a quasi-static particle (in the rotating frame) at different positions along the axis. The modeled particle experiences a focusing (non-zero) force towards the rotational axis up to a 12 s timepoint, promoting particle aggregation. 

### 2.4. Tea Leaf Settling Analysis

A schematic of the experimental setup is presented in Figure 1b. The setup consists of a vertical cylinder with a radius of $R_{0}=15$ (mm), where the base inclinations θ range from −20 to 40 degrees. The cylinders were fabricated using an SLA 3D Printer (AnyCubic Photon, ANYCUBIC Technology, Shenzhen, China). Prior to the experiment, the cylinder is filled with a suspension of tea leaves to a height of H=40 (mm), which corresponds to the numerical study domain depicted in Figure S2. The rotation of the vessel is driven by a stepper motor controlled by an Arduino microcontroller (Arduino, Ivrea, Italy). The cylinder with quiescent fluid is spun for 15 s at a rate ranging from 30 to 80 (RPM). After rotation, the cylinder is immobilized, and the particle evolution is studied. The motion and spatial distribution of particles are recorded using a digital Dino-Lite microscope (Dino-Lite, Hsinchu, Taiwan) mounted vertically above the rotational cell, as shown in Figure 1b. The grayscale video files are processed using a Processing Toolbox in MATLAB to analyze the post-rotation distribution of particles in the cell.

### 2.5. Tea Leaf Particle Characterization

An experimental setup was utilized to evaluate the size and density of tea leaves, which are crucial parameters for the numerical model. The size of the wetted tea leaf particles is estimated using a gauged microscope slide. Subsequently, the sedimentation time of the particles t was measured in a graduated cylinder with a known height of h=0.353 (m). Assuming particle sedimentation in the Stokes drag regime, the effective density of particles can be deduced using

(20) $$\rho_{p}=\frac{9\mu_{f}h}{2r_{p}^{2}gt}-\rho_{f}$$

where $\mu_{f}$ is the fluid viscosity, $\rho_{f}$ is the fluid density, g is gravitational acceleration, and $r_{p}$ is the effective particle radius. The average particle Reynolds number is 4.8 in the experimental conditions, suggesting that this equation is valid for these conditions. The measured and processed values for effective hydrodynamic particle radius and density are shown in Table S2. Figure S4 subsequently plots the effective density measured for particles of different radii. A linear trendline is fitted to the data as shown in Figure S4 to estimate particle density used in the numerical model (Table S1). 

To estimate the coefficient of friction, the angle of friction Θ was measured. The friction angle represents the maximum angle at which a particle will begin sliding. In the experiment, tea leaves are released above a submerged inclined surface. It is observed that when the angle exceeds Θ=60°, most particles start to slide. The solid friction constant (sliding friction) can thus be approximated from the friction angle, with

(21) μ=tanΘ≈1.7 .

## 3. Results and Discussion

### 3.1. Experimental Study

We first examine the experimental system to highlight key particle aggregation features, with subsequent numerical studies examining fluid velocities and equilibrium positions. In the experimental study, a vessel is rotated for 15 s while recording with a top-down microscope camera (Figure 1b). We use tea leaf particles rather than spherical manufactured ones in this work as the final equilibrium position of the latter would be dictated primarily by gravitational potential, with particles settling at the lowest potential position; using irregularly shaped tea particles mitigates particle rolling and allows other stable equilibrium positions to be examined. Figure 1c–e shows the temporal evolution of tea leaf spatial arrangement and aggregations in representative experimental setups, including different bottom surface inclinations (θ=0°, 20°, and 40°) and corresponding rotational rates (80 RPM, 50 RPM, and 30 RPM). Tea leaf particles, initially randomly distributed (frame 1), are forced to the edge of the rotating vessel by centrifugal force in the rotating vessel during rotation (frame 2, see Video S1 in the Supplementary Materials). As the rotation terminates, the secondary flow in the fluid induces particle movement toward the center of the cavity base (frame 3). The tea leaves, being denser than water, settle at the bottom of the vessel where flow exerts forces along with the vessel geometry, ultimately determining the final equilibrium particle aggregation radius (frame 4). In the case of a flat-bottom vessel rotating at a relatively high rate, the conventional tea leaf paradox focusing is observed, with particles aggregating at a single focal point in the vessel center (Figure 1c). In contrast, a conical chamber (θ=20°) produces a ring-shaped particle arrangement (Figure 1d). Highlighting the interplay between gravitational and fluidic forces, the chamber with a yet steeper inclination of the bottom surface (θ=40°) does not exhibit particle aggregation in the center; instead, the particles remain at the edge of the vessel (Figure 1e). These examples highlight the versatility of particle aggregation modes in this system, showcasing a wide range of possible spatial arrangements, where these can be modified via tuning of the system geometry and actuation parameters.

**Figure 2:**
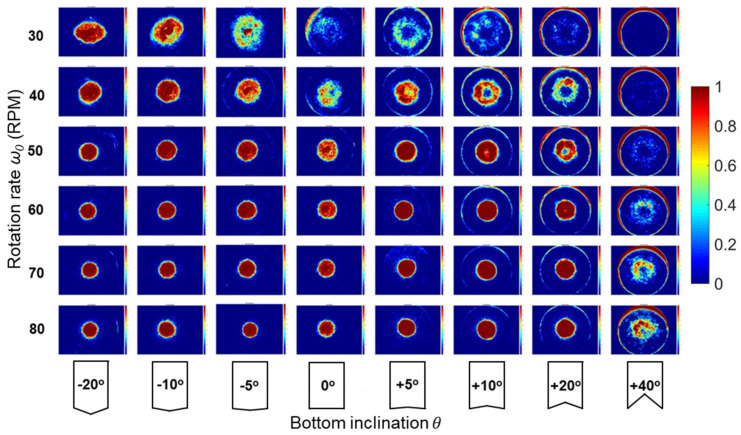
Tea leaf concentration profiles, settled in rotating vessels with bottom inclination θ=−20°,−10°,−5°,0°,+5°,+10°,+20°,+40° and initial angular velocity $\omega_{0}=30,\;40,\;50,\;60,\;70,\;80$ (RPM).

Figure 2 and Figure S5 (see Supplementary Materials) illustrate the experimental results of tea leaf particle distribution after settling under different conditions, where Figure 2 shows the cumulative particle settling positions for three repeats of each of the different combinations of rotational rates (30–80 rpm) and vessel geometries (−20° to 40°). Here, vessels with concave bases (5° to 40°) at low rotational rates tend to accumulate particles near the vessel wall (see Supplementary Video S2), where the low secondary flow velocities at low rotational speeds allow the particles to remain against the wall. As the rotational speed increases, the particle distribution transitions from the edge to aggregation in the center. This effect is amplified in convex cases (−20° to −5°) when the cylinder base is inclined downwards, where particles are preferentially translated inwards and aggregate at the center of the cylinder for a given rotational rate, with tighter focusing for higher rotational velocities. Interestingly, there is also a range of intermediate cases where ring-shaped focusing occurs, rather than at the vessel perimeter or center. Ring-shaped focusing behavior is primarily observed in the case of positive inclination angles. For lower rotational rates, elements of this are exhibited in the case of shallow-angle vessel bottoms (5° to 10°), whereas this feature is retained only for more steep positive angle vessel shapes (10° to 40°) as the rotation rate is increased. Together, these findings demonstrate the ability to achieve desired particle aggregations in each vessel as a function of these parameters. To examine these results in more detail, we use simulation results to examine fluid velocities in these geometries, with these used as inputs into subsequent modeling that combines viscous drag, inertial, lift and gravitational forces to predict equilibrium positions.

### 3.2. Computational Analysis

**Figure 3:**
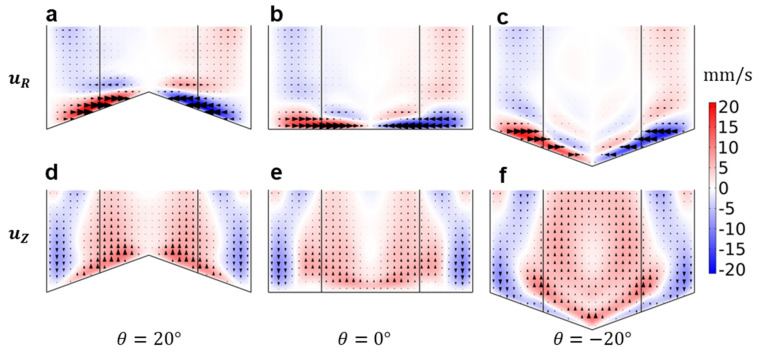
Vertical (**a**–**c**) and radial (**d**–**f**) components of the fluid flow in containers at $\omega_{0}=80$ RPM and 5 s after rotation termination.

Whereas the experimental analysis demonstrates the ability to generate different focusing patterns, computational analysis permits the interrogation of the secondary flow velocities that give rise to these effects. Fluid flow in the rotating cylindrical domains is studied using the finite element method (FEM) (see Figure S1, Supplementary Materials). The in-plane flow is characterized by a pair of vortices, located in close proximity to the bottom surface where there is a substantial lateral flow component along the base toward the rotational axis, where Figure S6 shows the fluid velocity 5 s after the rotation termination for different bottom topography and initial rotation rates. The velocity mapping also indicates the presence of secondary vortices, particularly in cases with higher initial velocities. Figure 3 shows the radial and vertical velocity components in the bottom part of the domain. Near the bottom boundary, radial flow is directed towards the axis of the container in all cases (Figure 3a–c), where this inward pointing flow is responsible for the tea leaf paradox focusing behavior. The vertical component ($u_{z}$) of flow along the bottom boundary, however, points upwards in convex geometries (positive angles) and downwards in concave ones (negative angles), whereas the vessel with a flat boundary exhibits far lower vertically oriented velocity magnitude across the lower boundary generally. In all cases, the radial velocity component is directed toward the center, with minimum velocities at the vessel outer walls and in the vessel center (and a maximum velocity magnitude between these positions). In both angled cases, it is notable that the vertical velocities against the bottom boundary are oriented such that there is a component oriented towards the vessel center (i.e., up a convex geometry or down a concave one). Together, these results indicate that particles will be universally drawn towards the vessel center regardless of vessel shape, though gravitational forces will act in opposition to fluid drag only in the case of convex (positively angled) vessel shapes.

**Figure 4:**
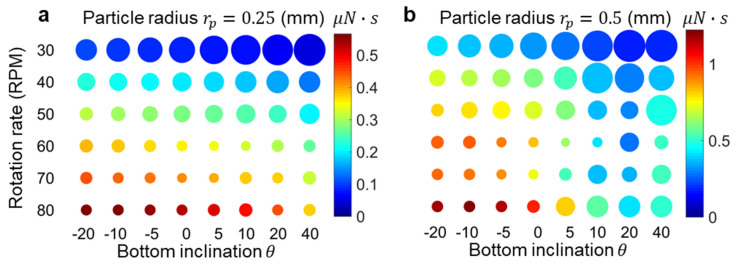
Numerical modeling results for (**a**) 0.25 and (**b**) 0.5 mm spherical particle settlement. The colormap indicates the average impulse $\bar{J_{x}}\;$ (nN·s) along the local coordinate x. The size of the circle corresponds to the stagnation radius, providing an estimate of the aggregation size.

Using the velocities obtained from modeling the different vessel shapes and rotation rates shown in Figure 2, the particle sedimentation is examined by analyzing forces acting on the particles, including gravitational, viscous drag and frictional interactions with the vessel bottom. Figure 4 provides a summary of the numerical modeling results depicting the tea leaf focusing phenomenon in different configurations. The transition of particles from the edge of the vessel to the center depends on the applied drag force. A higher focusing fluidic force promotes particles moving towards the center, leading to a tighter focusing spot. To quantify the efficiency of particle focusing in each case, we calculate the spatially averaged total impulse, denoted as

(22) $$\bar{J_{x}}=\frac{1}{R_{0}}\iint^{\;}F_{x}\left(r,t\right)dtdR,$$

where $R_{0}$ is the vessel radius. Each case in Figure 4 is color-coded to indicate the total impulse. Generally, cases with higher rotational rates exhibit larger averaged total impulse values. The focusing behavior is further quantified by the confining radius, represented in Figure 4 as the circle size. The confining radius represents the stagnation point location, which is the closest distance from the rotational axis where the force $\tilde{F_{X}}$ overcomes solid friction. It is observed that an increase in rotational speed generally leads to a tighter focusing spot, which is consistent with the experimental findings, where higher velocities can overcome frictional boundary interactions. Interestingly, vessels with smaller inclination angles tend to exhibit more compact focusing, especially for larger particles, where this effect is less pronounced for smaller particles.

### 3.3. Discussion and Outlook

The flow around a stationary particle can be characterized by the particle Reynolds number

(23) $$Re_{p}=\frac{2\rho_{f}u_{\tau }r_{p}}{\mu_{f}},$$

where $\mu_{f}$ is the fluid viscosity, $\rho_{f}$ is the fluid density, $r_{p}$ is the effective particle radius, and $u_{\tau }$ is the fluid velocity in the xz-plane (see coordinate system in Figure S2). The Reynolds number at the stagnation point in computational analysis is found to be, on average, 1.7, validating the assumption of Stokes drag. It is important, however, to note that the actual shape of tea leaves is complex and irregular, resembling small ragged-edged plates. The irregular shape of the particle results in a nonuniform drag constant as a function of orientation. Additionally, smaller, potentially more rounded sedimented particles may exhibit rolling. Conversely, flatter sedimented particles may experience a larger lift force in a shear flow, deviating from the predictions of the spherical approximation, although this same force (along with gravity) will tend to align particles flat against the bottom boundary. Nevertheless, despite the potential shape variations of the tea leaves, a particle model based on an effective hydrodynamic particle radius is found to align with the experimental findings, with tighter focusing in the case of higher secondary flow velocities, with some particles aggregating at intermediate positions in the case of positively angled walls. The base inclinations selected in this study are chosen in order to investigate the range of particle aggregation patterns, including what occurs in the vessel center, at the vessel perimeter, and in a ring at an intermediate distance. Smaller angles, e.g., θ<−20°, for instance, would not yield unique aggregation behaviors beyond those observed, with convergence in particle aggregation behavior yielding aggregation solely in the channel center for the negative angles tested.

**Figure 5:**
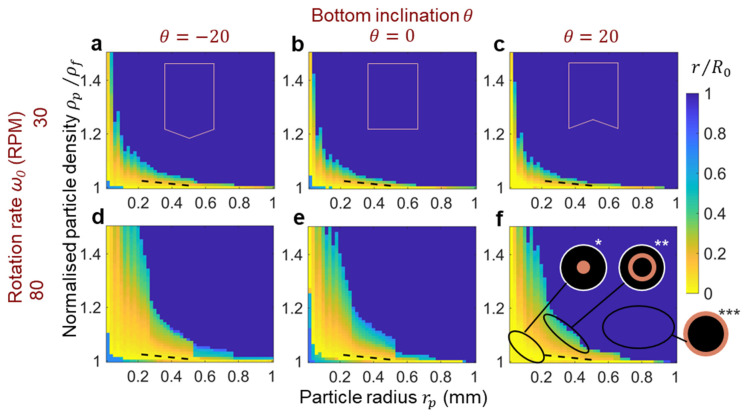
Calculated focusing spot radius $r/R_{0}$ as a function of particle density and size for vessels with bottom inclination angles (**a**,**d**) θ=−20°, (**b**,**e**) θ=0°, (**c**,**f**) θ=20°, and rotation rates (**a**–**c**) $\omega_{0}=30$, (**d**–**f**) $\omega_{0}=80$ (RPM). The pink boxes schematically illustrate the vessel shape. The dashed line corresponds to the experimentally estimated tea leaf properties. The inserts in (**f**) schematically show the particle aggregation modes: (*) focusing in the center, (**) ring-shaped aggregation, and (***) aggregation at the edge of the vessel.

The developed model can be further extended to predict the focusing behavior of particles with varying density and size. Figure 5 demonstrates the results of this parametric analysis, where the size of the focusing spot is estimated based on the stagnation radius (given by $r/R_{0}$, where values closer to 0 indicate tighter focusing). Each subfigure in this parametric analysis represents a contour plot of the results of computational analysis for particle settling in vessels for various combinations of base geometry, rotation rate and particle properties. The data points in the subfigures correspond to these modeling scenarios, where the focusing spot size is estimated numerically based on the given particle radius and density for a given combination of particle density and radius. All distributions exhibit a consistent pattern, showing that tight focusing occurs for either small-sized or less dense particles. Conversely, larger and heavier particles are expelled to the edge of the vessel. However, very small and low-density particles are swept to the edge as well due to being prone to follow the recirculating flow (i.e., if θ=−20°, 0°). Additionally, it is observed that an increase in the inclination angle of the bottom surface somewhat diminishes the overall focusing effect for a given rotation speed. Likewise, the rotation speed impacts the focusing size where a higher speed is necessary for larger and heavier particles to achieve similar focusing. The dashed line corresponds to the tea leaf properties, where higher speeds increase the proportion of particles that aggregate in the center for any given vessel inclination geometry. Notably, possible stagnation conditions exhibit a threshold of approximately halfway along the vessel bottom, where this is the location where the maximum radial velocities occur. Indeed, this accords with the experimental results in Figure 2, where ring-shaped aggregation typically occurs at radii at least half-way up the vessel base.

While our modeling approximates hydrodynamic drag interactions using a spherical particle approximation, using a hydrodynamically equivalent particle radius to approximate the behavior of non-spherical particles, it is essential to highlight the complexities introduced by non-spherical particle shapes commonly encountered in practical applications. Shape irregularity complicates the modeling approach, with particle orientation and particle rotation being complicating factors. Future work may seek to examine particle interactions at a more granular level while incorporating more complex particle geometries and accounting for additional hydrodynamic effects arising from non-spherical shapes. In addition to our examination of vessel geometry and rotational velocities, the interaction between particles and the vessel surface is another critical parameter that can impact sedimentation patterns. Particles with non-spherical shapes may have a different tendency to slip or roll along the inclined vessel bottoms of different roughness, leading to variations in their final positions. In conclusion, while our current study provides valuable insights into sedimentation under rotational flows, particle shape and slipping factors are crucial for future research to explore. These considerations could lead to a more comprehensive understanding of the complex interplay of factors affecting sedimentation behavior in various vessel geometries and rotational conditions.

## 4. Conclusions

This study presents a comprehensive investigation of particle sedimentation and aggregation in axisymmetric rotating vessels featuring secondary flow. The study reveals versatile particle aggregation patterns, including focused, ring, and edge aggregation formations, depending on the vessel geometry and rotational parameters. The results highlight the importance of careful control of the vessel design and operating conditions in achieving specific particle aggregation modes. The findings also demonstrate the applicability of utilizing these parameters in the context of the developed model for predicting particle focusing with varying particle properties. Overall, this work contributes to the understanding of particle behavior in rotating systems and provides insights for designing efficient and controlled particle manipulation processes in fields such as biotechnology, chemical engineering, and materials science. 

## Data Availability

Data underlying the results presented in this paper are not publicly available at this time but may be obtained from the authors upon reasonable request.

## Supplementary Materials

The following supporting information can be downloaded at: https://www.mdpi.com/article/10.3390/mi14112024/s1. Table S1. Simulation parameters. Table S2. Experimental particle radius and measured density. Table S3. Lateral force compensation factor on a stationary spherical particle in a shear field. Figure S1. Schematic diagram demonstrating the fluid domain in a cylindrical container with a radius *R*_0_, and height H in cylindrical coordinates (*R*, *Z*, *φ*). (a) Base geometries with concave inclinations (pointing upwards) are defined with a positive angle *θ*, (b) geometries with convex inclinations (tapered downwards) are defined with a negative angle *θ*. (c) Schematic diagram of the cross-section. (d) Forces acting on a swirling particle. Figure S2. (a) Computational mesh convergence study (b) Computational mesh. Figure S3. (a–c) Velocity components in the xz-plane near the bottom of the vessel 5 s after rotation termination. (d) The resulting force acting on the 0.375 um particle over time. The initial rotation rate is 80 RPM, *θ* = 0. Figure S4. Effective tea-leaf density for particles of different radii. The data is fitted with a least-squares linear trendline overlaid. Figure S5. Radial density distribution of settled tea leaf particles in vessels with various bottom surface inclinations and rotational rates. Figure S6. In-plane fluid velocity 5 s after the rotation stopped. The streamlines visualize local flow direction. Initial angular velocity is 30 RMP (**a**–**c**), 80 RPM (**d**–**f**). The base inclinations here are θ=20° (a,d), θ=0° (b,e), θ=−20° (c,f). Video S1: Experimental results for distinct tea leaf patterns including dot, ring, and edge particle aggregation governed by the vessel shape and rotation parameters. Video S2: Tea leaf focusing performance of vessels of different bottom inclination and rotation rate.
