# Supplementary material for: Thomson–Einstein’s Tea Leaf Paradox Revisited: Aggregation in Rings
Source: Micromachines (Basel). 2023 Oct 30;14(11):2024. doi: 10.3390/mi14112024 (PMC10672922; doi:10.3390/mi14112024)
Supplement: Supplementary file 1 [file micromachines-14-02024-s001.zip › micromachines-2669509 - Supplementary material.pdf]

micromachines

Supplementary Materials

Supplementary tables

Table S1. Simulation parameters.

**Water** at 21℃ [14]				
Density	ρ_f	997 kg m-3		
Dynamic viscosity	μ_f	0.00089 Pa s		
**Tea leaf particle (fitted data)**		1	2	3
Particle radius	r_p	0.25 mm	0.375 mm	0.5 mm
Particle density	ρ_p	1023 kg m-3	1015 kg m-3	1007 kg m-3

Table S2. Experimental particle radius and measured density.

r_p, mm	t, s	ρ_p, kg· m^{-3}
0.24	73.7	1032
0.52	71.2	1005
0.48	43.1	1011
0.37	46.9	1019
0.41	66.7	1010
0.27	112.3	1015
0.36	70.6	1013
0.36	69.3	1013
0.22	108.4	1023
0.50	70.8	1005
0.52	50.4	1007
0.44	46.4	1012
0.27	71.9	1026
0.26	108.4	1017
0.39	54.0	1015
0.37	61.3	1014

Table S3. Lateral force compensation factor on a stationary spherical particle in a shear field.

z/r_p	F_x^{s*}
∞	1
10.0677	1.0587
3.7622	1.1671
2.3524	1.278
1.5431	1.4391
1.1276	1.616
1.0453	1.6682
1.005004	1.6969
1.003202	1.6982
1	1.7005

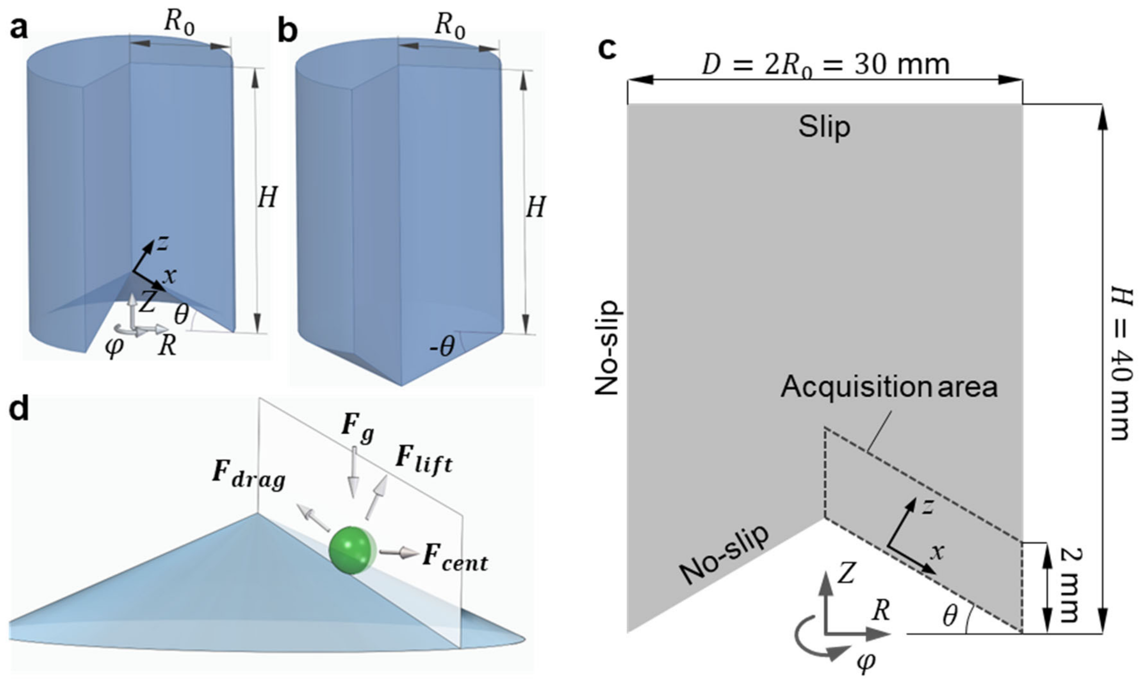

Figure S1. Schematic diagram demonstrating the fluid domain in a cylindrical container with a radius R_0, and height H in cylindrical coordinates (R, Z, φ). (**a**) Base geometries with concave inclinations (pointing upwards) are defined with a positive angle θ, (**b**) geometries with convex inclinations (tapered downwards) are defined with a negative angle θ. (**c**) Schematic diagram of the cross-section. (**d**) Forces acting on a swirling particle.

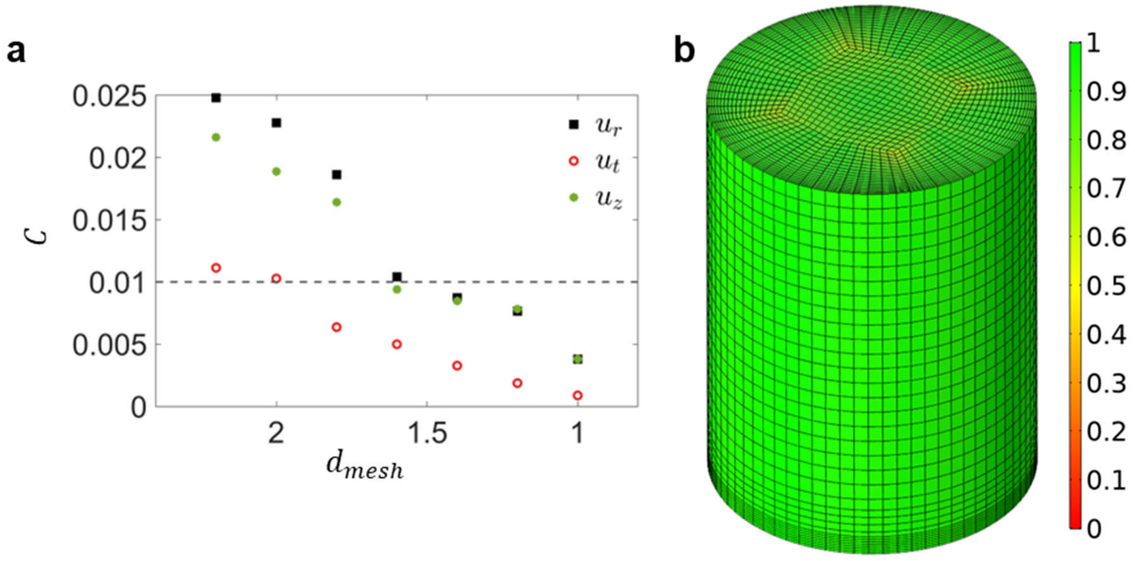

Figure S2. (**a**) Computational mesh convergence study (**b**) Computational mesh.

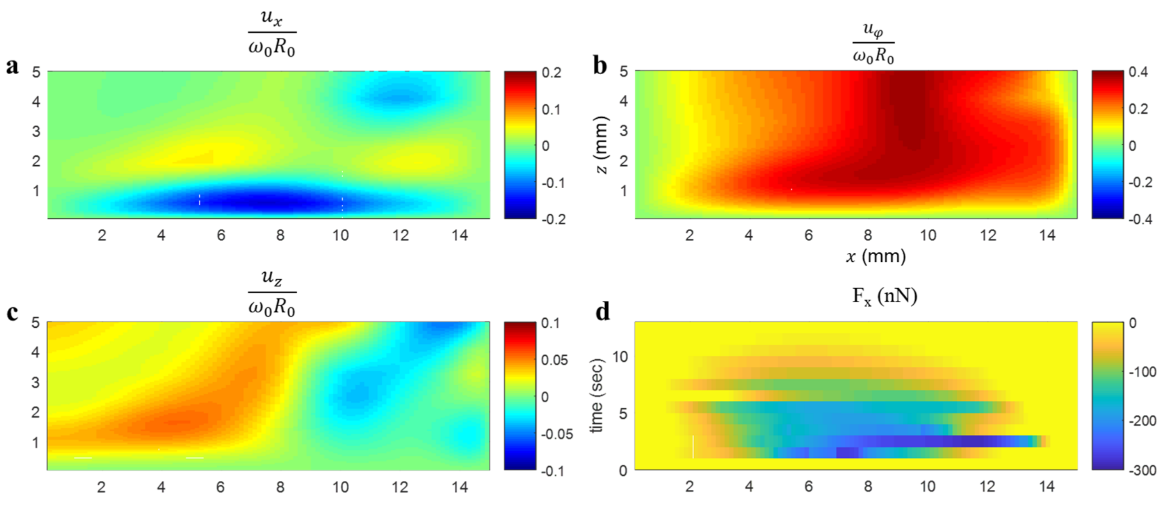

Figure S3. (**a**–**c**) Velocity components in the xz-plane near the bottom of the vessel 5 s after rotation termination. (**d**) The resulting force acting on the 0.375 um particle over time. The initial rotation rate is 80 RPM, $\theta = 0$.

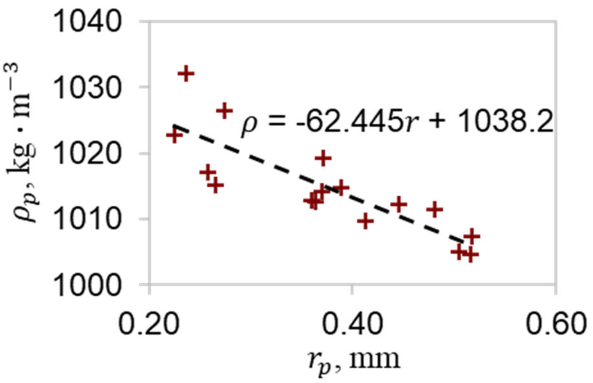

Figure S4. Effective tea-leaf density for particles of different radii. The data is fitted with a least-squares linear trendline overlaid.

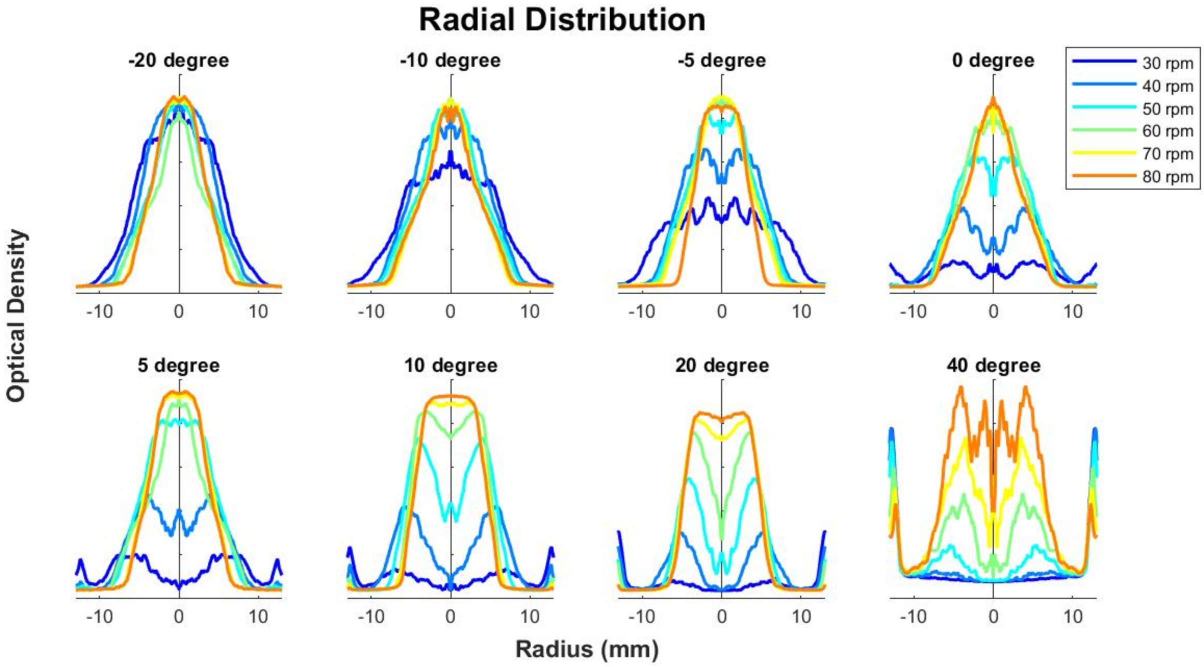

Figure S5. Radial density distribution of settled tea leaf particles in vessels with various bottom surface inclinations and rotational rates.

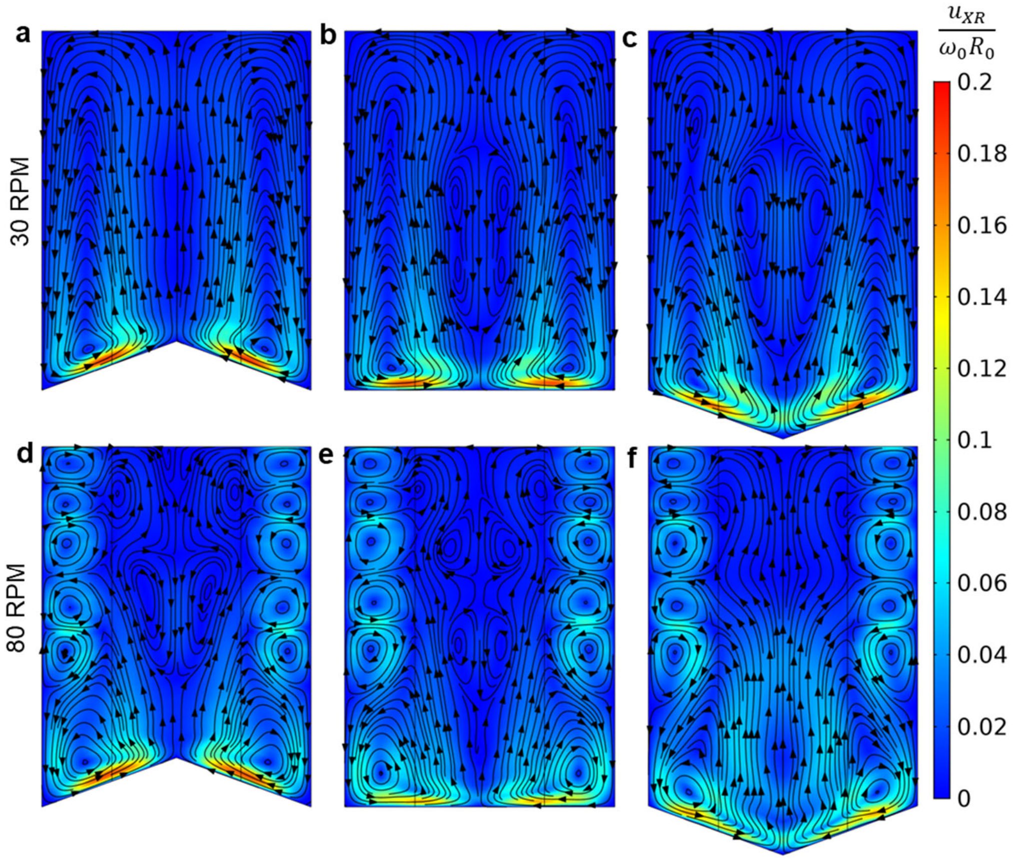

Figure S6. In-plane fluid velocity 5 seconds after the rotation stopped. The streamlines visualize local flow direction. Initial angular velocity is 30 RMP (**a**–**c**), 80 RPM (**d**–**f**). The base inclinations here are $\theta = 20°$ (**a**,**d**), $\theta = 0°$ (**b**,**e**), $\theta = -20°$ (**c**,**f**).
